# Supplementary material for: Liver-derived cell lines from cavefish Astyanax mexicanus as an in vitro model for studying metabolic adaptation
Source: Sci Rep. 2022 Jun 16;12:10115. doi: 10.1038/s41598-022-14507-0 (PMC9203785; doi:10.1038/s41598-022-14507-0)
Supplement: Supplementary file 1 — Supplementary Figures. [file 41598_2022_14507_MOESM1_ESM.docx]

**Supplementary figures**

**Supplementary Figure 1**: The graphs depict the standard curves for determining ALT enzyme activities using standard colorimetric assay.

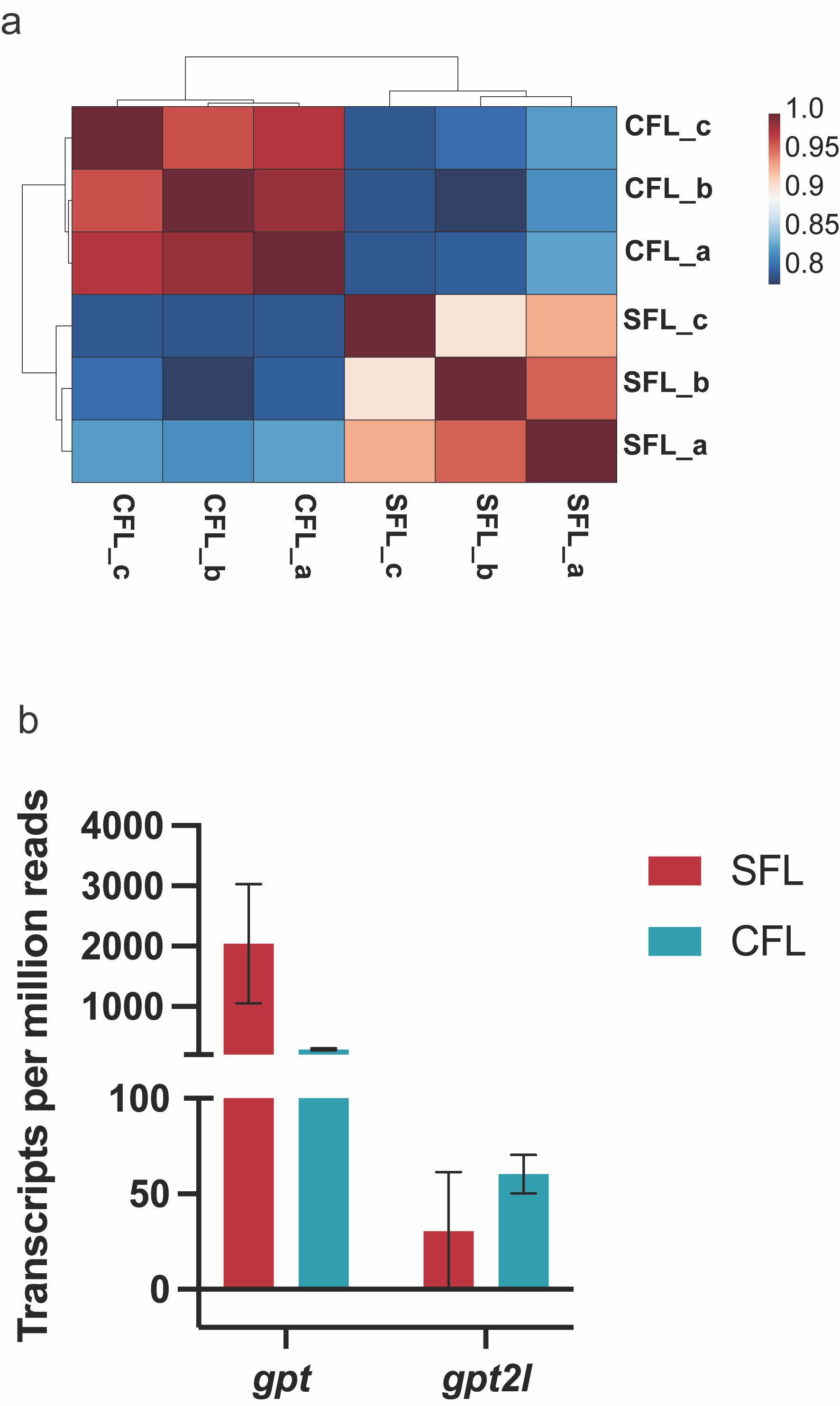


**Supplementary Figure 2**: (a) Spearman correlation coefficient heatmap for all cell line transcriptomes. (b) The graph shows expression levels of ALT genes (annotated as Glutamic--Pyruvic Transaminase or *gpt* on Ensembl).


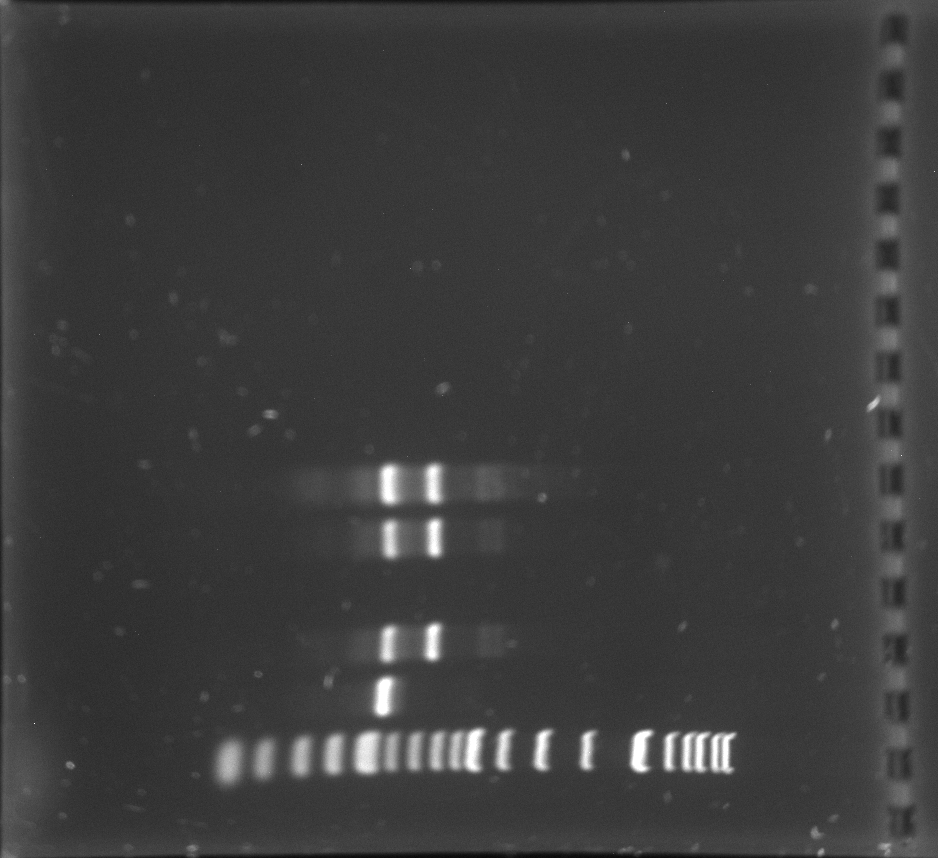


**Supplementary Figure 3:** Original picture for the agarose gel represented in Figure 2d (from left to right: muscle derived Pachón cells (not part of this study); liver derived Pachón cells; empty; male Pachón; female Pachón;

1 kb plus DNA marker).
