# Supplementary material for: Liver-derived cell lines from cavefish Astyanax mexicanus as an in vitro model for studying metabolic adaptation
Source: Sci Rep. 2022 Jun 16;12:10115. doi: 10.1038/s41598-022-14507-0 (PMC9203785; doi:10.1038/s41598-022-14507-0)
Supplement: Supplementary file 2 — Supplementary Legends. [file 41598_2022_14507_MOESM2_ESM.docx]

**Liver cell line marker genes**

The supplementary table 1 give the expression levels of the genes represented in the heatmap in Figure 4f which collectively make the marker genes for the *Astyanax* cell lines.
